# Working Memory Performance, Pain and Associated Clinical Variables in Women With Fibromyalgia

**DOI:** 10.3389/fpsyg.2021.747533

**Published:** 2021-10-21

**Authors:** Antonio Gil-Ugidos, Dolores Rodríguez-Salgado, Marina Pidal-Miranda, Noelia Samartin-Veiga, Montse Fernández-Prieto, Maria Teresa Carrillo-de-la-Peña

**Affiliations:** ^1^Brain and Pain (BaP) Laboratory, Department of Clinical Psychology and Psychobiology, University of Santiago de Compostela, Santiago de Compostela, Spain; ^2^Genetics Group, GC05, Instituto de Investigación Sanitaria de Santiago (IDIS), Santiago de Compostela, Spain; ^3^Grupo de Medicina Xenómica, Center for Research in Molecular Medicine and Chronic Diseases (CiMUS), Universidade de Santiago de Compostela (USC), Santiago de Compostela, Spain; ^4^U711, Centro de Investigación en Red de Enfermedades Raras (CIBERER), Santiago de Compostela, Spain

**Keywords:** fibromyalgia, cognitive dysfunction, pain threshold, working memory, health status, sleep dysfunction, fatigue

## Abstract

Working memory (WM) is a critical process for cognitive functioning in which fibromyalgia (FM) patients could show cognitive disturbances. Dyscognition in FM has been explained by interference from pain processing, which shares the neural substrates involved in cognition and may capture neural resources required to perform cognitive tasks. However, there is not yet data about how pain is related to WM performance, neither the role that other clinical variables could have. The objectives of this study were (1) to clarify the WM status of patients with FM and its relationship with nociception, and (2) to determine the clinical variables associated to FM that best predict WM performance. To this end, 132 women with FM undertook a neuropsychological assessment of WM functioning (Digit span, Spatial span, ACT tests and a 2-Back task) and a complete clinical assessment (FSQ, FIQ-R, BDI-1A, HADS, PSQI, MFE-30 questionnaires), including determination of pain thresholds and tolerance by pressure algometry. Patients with FM seem to preserve their WM span and ability to maintain and manipulate information online for both visuospatial and verbal domains. However, up to one-third of patients showed impairment in tasks requiring more short-term memory load, divided attention, and information processing ability (measured by the ACT task). Cognitive performance was spuriously related to the level of pain experienced, finding only that pain measures are related to the ACT task. The results of the linear regression analyses suggest that sleep problems and fatigue were the variables that best predicted WM performance in FM patients. Future research should take these variables into account when evaluating dyscognition in FM and should include dynamic measures of pain modulation.

## Introduction

Fibromyalgia (FM) is a chronic pain syndrome without a fully known organic etiology, characterized by widespread musculoskeletal pain, stiffness, fatigue, non-restorative sleep, and cognitive dysfunction (Wolfe et al., 2010). Recent research has shown the importance of cognitive symptoms in patients with FM (Dick et al., 2008; Seo et al., 2012; Tesio et al., 2014; Coppieters et al., 2015; Gelonch et al., 2018; Pidal-Miranda et al., 2018). They very often complain about cognitive difficulties in memory, attention or ability to concentrate, commonly called “fibro fog,” which they report as even more disturbing than pain (Williams et al., 2011). Dyscognition is the term used to refer to both the subjectively and objectively determined cognitive symptoms that worsen the disability generated by the syndrome itself and that have a significant impact on the day-to-day life and functioning of individuals (Katz et al., 2004; Ambrose et al., 2012; Sallinen and Mengshoel, 2018).

Although the presence of cognitive complaints is clearly noted in patients with FM, their specific objective cognitive symptoms are less well established (Wu et al., 2018). Working memory is a cognitive function critical for the attentional and executive functioning in which objective alterations have been found (Park et al., 2001; Dick et al., 2008; Cánovas et al., 2009; Kim et al., 2011; Seo et al., 2012; Tesio et al., 2014; Coppieters et al., 2015; Gelonch et al., 2018; Pidal-Miranda et al., 2018) and has been proposed as central to dyscognition in FM (Ambrose et al., 2012); however, other results have failed to find WM disturbances (Walitt et al., 2016; Kratz et al., 2020). Moreover, it has been overlooked that WM is a multicomponent construct of memory, reserved for the retention and manipulation of small amounts of new or retrieved information (Baddeley and Hitch, 1974). The updated version of the Multicomponent Model of working memory (Baddeley, 2000) considers that WM activity is divided into four subsystems: the Central Executive system, which involves attention control and organization of cognitive resources and their distribution; the Phonological Loop, which involves the storage of phonological information and the processes of articulatory control; the Visuospatial Sketchpad, which involves recording and storage of spatial information associated with visual information; and the Episodic Buffer, which manages the establishment of episodic long-term memory. Therefore, studies are necessary to clarify the status of FM patients in WM by attending to its different components.

Dyscognition in FM has been explained as interference from pain processing in cognition, as both share neural resources, e.g., the cingulate cortex and supplementary motor area (Buhle and Wager, 2010; Schmidt-Wilcke et al., 2014; Sturgeon et al., 2015), but data are not conclusive. It is certain that some previous studies have found a relationship between cognition and pain threshold or tolerance ratings, both for experimentally induced pain and for behavioral pain indices. In fact, the existing evidence supports the idea that detection and processing of nociceptive stimulation could affect WM ability in FM patients (Munguía-Izquierdo et al., 2008; Buhle and Wager, 2010; Sanchez, 2011; Hood et al., 2013; Nakae et al., 2013; Coppieters et al., 2015; Sturgeon et al., 2015; Boselie et al., 2016). It has been suggested that attentional aspects related to WM, which are crucial for good task performance, could be affected by pain (Moore et al., 2012, 2019), and measures of endogenous pain inhibition could be used to predict cognitive performance of patients with FM (Ickmans et al., 2015). Nevertheless, in some studies such cognitive alterations were only observed in relation to application of acute pain (Hood et al., 2013; Boselie et al., 2016), even with strong stimulation only (Coppieters et al., 2015), and other studies did not support the connection between nociception and cognition (Suhr, 2003; Etherton, 2014; Gálvez-Sanchez et al., 2018). Thus, the link between pain and cognition remains unclear. In particular, there is not enough data about the relationship between nociception and WM performance, a critical process for cognitive well-functioning.

Likewise, the relationship between WM performance and other clinical symptoms apart from pain in FM patients is not clear. It has been suggested that depressed mood (Seo et al., 2012; Gelonch et al., 2017, 2018; Wu et al., 2018), anxiety (Munguía-Izquierdo et al., 2008; Seo et al., 2012; Wu et al., 2018), fatigue (Suhr, 2003) and sleep disturbance (Ceko et al., 2012; Aasvik et al., 2018) may be relevant to explain dyscognition in FM (Gelonch et al., 2013; Pidal-Miranda et al., 2018). However, other studies confirmed the presence of dyscognition even after ruling out the effect of those symptoms (Dick et al., 2008). Knowing the effect of all these variables on the cognitive status of FM patients, particularly in critical cognitive processes such as WM, may be useful in addressing the impact of dyscognition associated to FM.

The objectives of our study were (1) to clarify the WM status of patients diagnosed with FM and its relationship with nociception, and (2) to determine the clinical variables associated to FM that best predict WM performance. For this purpose, we assessed FM patients with a variety of tests selected to cover the main components of WM, and we also examined their level of pain experienced (i.e., pressure pain thresholds and tolerance). Relationship between these variables were assessed, and performance on working memory tasks of high and low pain threshold groups was compared. Finally, we analyzed which of a series of clinical variables self-reported by patients (pain, fatigue, sleep dysfunction, morning stiffness, functional state, health status, depression, anxiety and cognitive complaints) are the best predictors of WM performance. In line with the evidence reported above, we hypothesized that FM patients with lower pain thresholds (greater pain sensitivity) would perform less well in WM tasks. Furthermore, in relation to the clinical variables, we expected that greater severity of FM symptoms and cognitive complaints would be associated with objective poorer performance in WM tasks.

## Materials and Methods

### Patients and Procedure

The study participants were 132 women (mean age = 50.53 years; SD = 8.71) who were already taking part in a clinical trial to assess the effect of transcranial electrical stimulation on fibromyalgia. This clinical trial was conducted in Galicia (Spain) in the period from May 2017 to November 2018. It followed the Declaration of Helsinki, was preregistered at http://www.encepp.eu/(Register number: 24294) and approved by the Research Ethics Committee of Galicia (code: 2014/488).

The first contact with participants was by phone; those who agreed to participate were scheduled for a first evaluation session, where we obtained the informed consent and confirmed the fulfillment of the selection criteria.

The following inclusion criteria were applied: age between 25 and 65 years, diagnosis of fibromyalgia (usually made by the family doctor and confirmed by a rheumatologist) and compliance with American College of Rheumatology (ACR) criteria for fibromyalgia (Wolfe et al., 1990, 2010). The exclusion criteria were as follows: presence of immune pathology or comorbidities that could explain the main symptomatology of FM; presence of brain damage, dementia or Parkinson’s disease; presence of psychiatric disorders (other than anxiety or depression); and other risk factors or aspects related to the trial intervention (as this consisted of transcranial brain stimulation, we excluded participants with previous history of seizures-epilepsy, use of anticonvulsant treatment, or any change in the recent medication pattern).

The data analyzed were obtained in the pre-treatment evaluation session of the clinical trial. This session consisted of a complete clinical assessment that included a semi-structured interview and a series of self-report questionnaires to assess the health status relative to the core symptoms of FM, pressure algometry at tender points to measure pain threshold and tolerance, and a WM neuropsychological assessment (see description in *Measures* section). For ethical reasons, withdrawal of their analgesic treatments was not requested during the study. The evaluations were conducted in the *Brain and Pain Laboratory* of the University of Santiago de Compostela and in health centers/hospitals in Galicia (NW Spain).

### Measures

#### Pressure Algometry

A trained researcher evaluated the pain threshold and tolerance of the patients by using a pressure algometer (Wagner Force One, Model FDI) at the 18 tender points proposed by the ACR (Wolfe et al., 1990). Pain threshold was defined as the minimum applied force (kg/cm^2^) that induced pain. A point was defined as positive when the participant felt pain at pressure lower than 4 kg/cm^2^, as it has been established that in healthy women pain begins to be perceived at pressure greater than this level (Wolfe et al., 1990). For each participant, we considered the total number of positive tender points, and the average threshold at the 18 tender points. Pressure pain tolerance (kg/cm^2^) was defined as the maximum pain-pressure value that was born at each point.

#### Self-Reported Scales to Assess Fibromyalgia Symptoms (Spanish Validated Versions)

Visual Analogical Scales (VAS) were created *ad hoc* to assess the clinical status of the participants. The scales consist of a set of 10 cm long horizontal lines, scored from 0 to 10 (left end represented the best condition and the right the worst). The participants were asked to indicate their status in relation to the following variables: pain, functional state, morning stiffness, fatigue, mood, health status and non-restorative sleep.

The Revised Fibromyalgia Impact Questionnaire (FIQ-R; Bennett et al., 2009; Salgueiro et al., 2013) was administered to measure the functional disability and health status of FM patients. The FIQ-R is a self-reporting questionnaire of 21 items, scored on an 11-point numerical rating scale of 0–10, with 10 being “worst.” The scores are calculated for three domains: function (from 0 to 30), general impact (from 0 to 20) and symptoms (from 0 to 50). A total score (range from 0 to 100) was also considered. Higher scores are associated with greater disease severity and functional impact, and patients could be classified in mild effect (0–38), moderate effect (39–58) and severe effect (59–100).

The Fibromyalgia Survey Questionnaire (FSQ; Wolfe et al., 2011; Carrillo-de-la-Peña et al., 2015) was used to assess FM symptoms. The FSQ is based on the diagnostic criteria proposed by Wolfe et al. (2010) and includes the Symptom Severity Scale (SSS) and the Widespread Pain Index (WPI). The SSS considers three key symptoms (fatigue; cognitive problems in attention, concentration or memory; and non-restorative sleep), assessed on a scale of 0–3 (0 = not present to 3 = extreme). In addition, the SSS assesses abdominal pain, depression and headache, determined as present (1) or not present (0). The SSS score ranges from 0 to 12. The WPI score indicates the number of body areas with pain reported by the patient (from 0 to 19). The SSS and WPI scores and the total FSQ score (sum of WPI and SSS) were considered in the subsequent data analysis.

The Beck Depression Inventory (BDI-1A; Sanz and Vázquez, 1998; Beck et al., 1961) was used to evaluate the severity of depressive symptoms. The BDI-1A is composed by 21 items representative of symptoms such as sadness, feelings of failure, pessimism, suicidal desires, etc. Each item is answered by the participants on a 4-point scale, ranging from 0 to 3. The total score (range from 0 to 63) was considered. Higher scores are associated with greater severity of depressive symptoms, and patients could be classified in lack of depression (0–13), mild depression (14–19), moderate depression (20–28) and severe depression (29–63).

The Hospital Anxiety and Depression Scale (HADS; Zigmond and Snaith, 1983; Rico et al., 2005) was used to assess the presence of anxiety and depression symptomatology. The HADS is a 14-item Likert type scale (response range between 0–3), enabling patients to describe the feelings they had experienced during the previous week. The total score ranges from 0 to 42, and higher scores are associated with greater severity of symptomatology. It is divided into two 7-item subscales (score range 0–21 for each), one for anxiety and one for depression. For both scales, if the score is higher than 8, impairment is considered possible, and if it is higher than 11, it is considered probable.

Pittsburgh Sleep Quality Index (PSQI; Buysse et al., 1989; Royuela and Macías, 1997) was used to assess patients’ sleep quality and dysfunction in the last month. The PSQI is composed of 7 Likert type subscales related to sleep problems and rated between 0 (absence of difficulty) and 3 (maximum difficulty). The total score ranges from 0 to 21, and higher scores are associated with poorer quality of sleep. A total score of 5 would be the cut-off point, separating subjects who have good sleep quality from those who have poor sleep quality.

Everyday Memory Failures Questionnaire-30 (MFE-30; Lozoya-Delgado et al., 2012; Sunderland et al., 1984) was administered in order to evaluate subjective memory problems, and other cognitive complaints related to perceptual, linguistic and praxical processes. The MFE-30 consists of 30 Likert-type response items rated between 0 (never or almost never) to 4 (always or almost always). The total score (range from 0 to 120) was considered, and patients could be classified in optimal performance (0–7), normal performance (8–35), mild impairment (36–50) and moderate impairment (>50).

#### Working Memory Neuropsychological Testing

**FIGURE 1:**
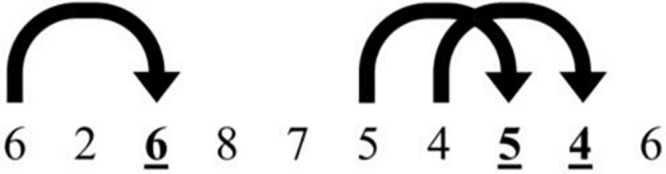
Example of target stimuli (underline numbers) in the visual 2-back task.

A visual 2-back task was designed using the PsychoPy software (Peirce, 2009), to evaluate the ability to maintain, monitor, manipulate and update information in WM. Patients were asked to monitor a sequence of digits (from 0 to 9) presented on a computer screen, and to press a button with the index finger of their dominant hand when a target stimulus appeared. The target stimulus was the digit identical to that presented two trials before (Figure 1). The task consisted of 200 trials, with 30% target stimuli. The number of correct answers, errors and omissions were recorded, along with the average response times associated with hits and errors.

The Digit Span Subtest of the Wechsler Memory Scale (WMS- III; Wechsler, 1997) was used to evaluate the phonological loop and the ability to manipulate verbal information in WM. Participants were asked to repeat chains of digits of increasing length in the given order in the *Forward* task and in reverse order in the *Backward* task. The number of correct items repeated and span scores (the maximum number of digits correctly produced) were obtained for the Forward and Backward tasks.

Spatial Span Subtest of the Wechsler Memory Scale (WMS-III; Wechsler, 1997) was used to evaluate aspects of the visuospatial agenda and the ability of the subjects to manipulate visuospatial information in WM. In the task, nine cubes were placed on a board. The examiner tapped the cubes in a specific order, starting with two blocks and gradually increasing the series. In the *Forward* part, participants were asked to repeat the observed sequence. In the *Backward* part, they had to repeat the sequence in reverse order. The number of correct items and span scores were obtained for the Forward and Backward tasks.

The Auditory Consonant Trigram Test (ACT; Brown, 1958; Peterson and Peterson, 1959) was used to evaluate short-term memory, divided attention and information processing capacity. This task consists of the verbal delivery of a series of consonant trigrams (three letters) at a rate of one letter per second. A three-digit random number was presented immediately after each trigram. Participants were asked to memorize the letters and then to count aloud, starting from the number provided, backward three at a time. They were asked to do this over time intervals of 0, 9, 18 and 36 s, with five trials for each time interval (a total of 20). The number of letters remembered in each trial was recorded and a total score calculated (between 0 and 60).

### Data Analysis

Means and standard deviations were used to describe the quantitative variables, while absolute frequencies and percentages were used for the qualitative measures.

Raw scores obtained from the WM tasks were converted, when possible, to age and education adjusted scaled scores according to normative data (Stuss et al., 1987; Anil et al., 2003; Peña-Casanova et al., 2009; Tamayo et al., 2012). Subsequently, scores that were at least 1.5 standard deviations below the mean were judged to be abnormally low and to suggest clinical significance.

A Pearson correlation test was performed to assess the relationship between pain threshold and WM scores. In addition, performance on working memory tasks of “High pain threshold patients” and “Low pain threshold patients” groups (the median pain threshold score was established as cut-off point) was compared using Student *t*-tests for independent samples. Moreover, data were analyzed by means of Bayesian approach to assess evidence for and against the effects. To describe the Bayes factors, we used the classification scheme of Lee and Wagenmakers (Quintana and Williams, 2018).

Linear regression analyses were applied to each of the WM scores of FM patients, using nociceptive (pain threshold and tolerance) and the self-reported clinical measures (SSS, WPI and Total Score of the FSQ; Function, General Impact, Symptoms and Total Score of the FIQ-R; Pain, Functional State, Morning Stiffness, Fatigue, Mood, Health Status and Non-restorative Sleep VAS; BDI-1A; HADS; PSQI; and MFE-30) as possible predictors.

Data were analyzed using the SPSS statistical package (v.24.0; IBM Corporation, Armonk, NY, United States).

## Results

### Sample Characteristics

The descriptive statistics corresponding to participants’ data relative to the demographic, clinical and self-reported measures to assess FM symptoms are summarized in Table 1 (the *n* varied in each task, due to missing data).

**TABLE 1:** Demographic, clinical and self-reported characteristics of fibromyalgia patients.

	*N*	Min–Max	Mean (SD)	Frequency (%)
Age (years)	132	25–66	50.53 (8.71)	
Education (years)	132	10–16	12.64 (2.41)	
Years from diagnosis	129	0–48	9.96 (7.99)	
Pain threshold (kg/cm^2^)	114	0.16–5.21	1.98 (1.01)	
Low	56	0.16–1.89		49.12
High	58	1.90–5.21		50.88
Pain tolerance	114	0.38–7.11	2.66 (1.45)	
Tender points	114	5–18	16.23 (3.09)	
FSQ:				
SSS (range 0–12)	131	0–12	7.44 (1.78)	
WPI (range 0–19)	131	3–19	11.81 (3.99)	
Total score (range 0–31)	131	10–28	19.24 (4.48)	
FIQ-R:				
Function (range 0–30)	131	3.33–30	18.72 (6.17)	
General impact (range 0–20)	131	0–20	12.34 (5.33)	
Symptoms (range 0–50)	130	10.5–50	36.32 (8.69)	
Total score (range 0–100)	130	19.67–99.67	67.43 (17.88)	
Mild effect (range < 39)	7	19.67–38.83		5.38
Moderate effect (range 39–59)	34	39.50–58.83		26.15
Severe effect (range 59–100)	89	59–99.67		68.46
VAS (range 0–10):				
Pain	126	1- 10	7.29 (1.88)	
Functional state	126	0–10	7.12 (2.26)	
Morning stiffness	126	0–10	7.59 (2.86)	
Fatigue	125	2–10	8.16 (1.83)	
Mood	126	0–10	5.71 (3.07)	
Health status	125	0–10	7.42 (2.58)	
Non-restorative sleep	126	0–10	7.76 (2.51)	
BDI-1A Total	132	0–49	21.82 (10.65)	
Lack of depression (range 0–13)	28	0–13		21.21
Mild depression (range 14–19)	32	14–19		24.24
Moderate depression (range 20–28)	40	20–28		30.30
Severe depression (range 29–63)	32	29–49		24.24
HADS Total	131	4–42	21.22 (7.28)	
Anxiety Scale Total	131	1–21	12.16 (4.21)	
Without anxiety (range 0–7)	18	1–7		13.74
Possible anxiety (range 8–10)	42	8–11		32.06
Probable anxiety (range 11–21)	71	12–21		54.19
Depression Scale Total	131	1–21	9.02 (3.95)	
Without depression (range 0–7)	50	1–7		38.17
Possible depression (8–10)	44	8–11		33.59
Probable depression (11–21)	37	12–21		28.24
PSQI Total	127	2–21	13.09 (4.37)	
Good sleep quality (range 0–5)	9	2–5		7.09
Poor sleep quality (range 6–21)	118	6–21		92.91
MFE-30 Total	131	2–111	53.36 (25.89)	
Optimal performance (range 0–7)	4	2–6		3.1
Normal performance (range 8–35)	32	8–35		24.81
Mild impairment (range 36–50)	23	36–50		17.83
Moderate impairment (>50)	72	50–111		55.81

*FSQ, Fibromyalgia Survey Questionnaire; SSS, Symptom Severity Scale; WPI, Widespread Pain Index; FIQ-R, Fibromyalgia Impact Questionnaire; VAS, Visual Analogical Scales; BDI-1A, Beck Depression Inventory; HADS, Hospital Anxiety and Depression Scale; PSQI, Pittsburgh Sleep Quality Index; MFE-30, Everyday Memory Failures Questionnaire (note that the *n* varied in each task, due to missing data).*

The mean number of years from patients’ diagnosis of FM was 9.96 (SD = 7.99). Their average pain threshold was 1.98 kg/cm^2^ (SD = 1.01), which is below the 4 kg/cm^2^ limit set by the ACR criteria, and their average tolerance was 2.66 kg/cm^2^. The scores on the VAS (range 0–10) showed that means of all measures were over the midpoint of the range (5 out of 10), and the symptom that manifested itself most intensely was fatigue (*M* = 8.16; SD = 1.83).

Regards measures of impact and severity of FM, the average total score on the FIQ-R showed a severe impact of 67.43 (out of 100), and an average score of 19.24 (out of 31) was obtained for the fibromyalgia symptomatology measured with the FSQ.

For the BDI-1A the average score was 21.82 points, which according to the normative data (Sanz and Vázquez, 1998) corresponds to a moderate degree of depression. Similarly, up to 54.54% of the participants scores were classified between moderate and severe depression, indicating a high presence of depressive symptomatology among the sample participants. For the HADS, the average score was 21.22, which according to the normative data (Rico et al., 2005) indicates a clinical problem. More specifically, up to 54.19% of participants are likely to have anxiety based on the anxiety scale scores and based on the depression scale up to 28.24% of participants are likely to have depression.

The average score obtained by the participants on the PSQI was 13.09, which is higher than the score of 5 established as the cut-off point between good and poor-quality sleep (Royuela and Macías, 1997). In fact, as many as 92.91% of participants reported poor sleep quality.

Finally, cognitive complaints were frequently reported in the MFE-30 by patients, with more than 70% of patients showing total scores between mild impairment and moderate impairment. Moreover, the average score was higher than 50 points, which according to available data (Lozoya-Delgado et al., 2012) indicates moderate or severe amnestic impairment with some impact on daily functioning.

### Working Memory Neuropsychological Performance

**FIGURE 2:**
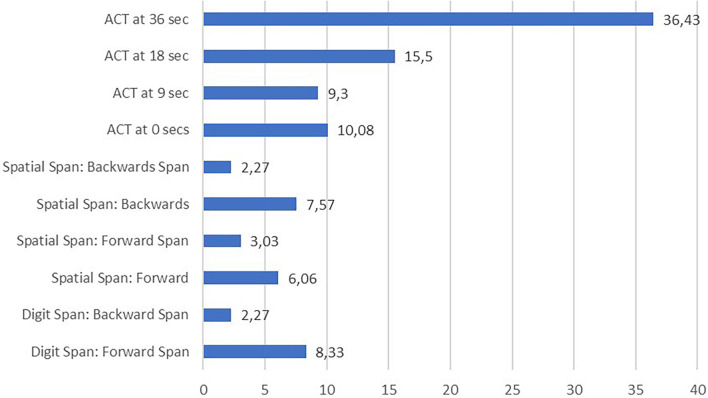
Percentage of fibromyalgia patients with deficient performance in working memory measures based on normative data (< 1.5 SD below normative mean).

The descriptive statistics corresponding to the measures from the different tasks used in the study to assess WM in FM patients are summarized in Table 2. Regarding the analysis of individual WM performance compared to normative data obtained from the general healthy population, Figure 2 shows the percentage of FM patients with impaired performance (*Z* ≤ −1.5) in the different WM measures.

**TABLE 2:** Descriptive statistics of patients performance on working memory tasks

	*N*	Min–Max	Mean (SD)
2-Back:			
Hits	119	6–57	41.78 (9.91)
Hits- RT (s.)	119	0.37–0.87	0.59 (0.12)
Errors	119	1–84	13.37 (12.99)
Errors- RT (s)	119	0.36–1.08	0.63 (0.14)
Omissions	119	2–55	18.92 (9.86)
Digit Span:			
Forward	132	3–16	8.34 (2.16)
Forward Span	132	3–9	5.74 (1.22)
Backward	132	2–11	5.3 (1.75)
Backward Span	132	2–7	4.18 (1.08)
Spatial Span:			
Forward	132	2–12	6.43 (1.97)
Forward Span	132	2–8	4.79 (1.15)
Backward	132	2–11	5.66 (1.79)
Backward Span	132	2–8	4.37 (1.04)
ACT at 0 s	130	0–15	14.62 (1.44)
ACT at 9 s	130	0–15	9.16 (3.35)
ACT at 18 s	130	1–15	6.74 (3.58)
ACT at 36 s	130	0–14	6.69 (3.43)
Total ACT Score	130	20–56	37.22 (8.65)

*RT, Response Time; ACT, Auditory Consonant Trigram Test.*

The ACT task had the highest percentage of patients with scores 1.5 SD below the normative mean: 36.43% at 36 s, 15.5% at 18 s, 9.3% at 6 s and 10.08% at 0 s. On the other hand, Digit and Spatial Span produced the smaller percentages of patients with scores 1.5 SD below the normative mean (2.27%).

### Working Memory Performance and Pain Threshold

Pearson correlation analysis to assess the relationship between pain threshold and WM scores were carried out. Only the correlation between pain threshold and ACT at 9 s was found to be significant (*r* = −0.205; *p* = 0.031). Using the Bayesian approach, correlation analyses indicated anecdotal evidence of no correlation with pain threshold for ACT at 9 s (BF_10_ = 1.187). It was also found anecdotal/moderate evidence of no correlation with pain threshold for all other WM variables.

Moreover, the results of the comparison of WM performance of FM patients’ subgroups of High Pain Threshold (>1.89 kg/cm^2^) and Low Pain Threshold (≤1.89 kg/cm^2^) using *t*-test for independent samples are presented in Table 3, including also the BF_10_ values obtained using the Bayesian approach. No significant differences between the subgroups were found for any of the WM performance scores using frequent statistics. In addition, we found anecdotal/moderate evidence in favor of an absence of difference in WM performance between the high and low pain threshold groups, based on Bayesian analyses.

**TABLE 3:** Results in working memory tasks for the “low pain threshold” and “high pain threshold” groups

Group					
WM Task	Low pain threshold *N* = 56 Mean (SD)	High pain threshold *N* = 58 Mean (SD)	*t*	*p*	*BF*_10_
2-Back Correct	42.13 (± 10.04)	42.39 (± 8.23)	0.144	n.s.	0.212
2-Back Correct: RT (s)	0.58 (± 0.11)	0.6 (± 0.12)	0.895	n.s.	0.300
2-Back Errors	14.51 (± 14.08)	14.26 (± 13.67)	–0.091	n.s.	0.211
2-Back Errors: RT (s)	0.62 (± 0.14)	0.63 (± 0.14)	0.658	n.s.	0.255
2-Back Omissions	18.45 (± 9.97)	18.44 (± 8.22)	–0.001	n.s.	0.210
Digit Span: Forward	8.25 (± 1.83)	8.41 (± 2.45)	0.403	n.s.	0.235
Digit Span: Forward Span	5.70 (± 1.08)	5.74 (± 1.4)	0.192	n.s.	0.218
Digit Span: Backward	5.18 (± 1.74)	5.21 (± 1.79)	0.086	n.s.	0.203
Digit Span: Backward Span	4.05 (± 1.09)	4.14 (± 1.1)	0.412	n.s.	0.216
Spatial Span: Forward	6.61 (± 1.78)	6.19 (± 2.04)	–1.164	n.s.	0.253
Spatial Span: Forward Span	4.88 (± 1.05)	4.69 (± 1.22)	–0.871	n.s.	0.231
Spatial Span: Backward	5.70 (± 1.92)	5.69 (± 1.83)	–0.019	n.s.	0.201
Spatial Span: Backward Span	4.39 (± 1)	4.38 (± 1.17)	–0.066	n.s.	0.202
ACT at 0 s	14.81 (0.61)	14.43 (2.12)	1.298	n.s.	0.470
ACT at 9 s	8.93 (3.37)	9.43 (2.96)	–0.833	n.s.	0.228
ACT at 18 s	6.40 (3.5)	6.53 (3.38)	–0.201	n.s.	0.202
ACT at 36 s	6.26 (3.5)	6.77 (3.28)	–0.798	n.s.	0.244
Total ACT Score	37.17 (± 7.44)	36.40 (± 8.89)	–0.495	n.s.	0.204

*WM, working memory; RT, response Time; ACT, Auditory Consonant Trigram Test.*

### Working Memory Performance and Clinical Variables

Results of the linear regression analyses performed for each of the recorded WM scores, using nociceptive (pain threshold and tolerance) and the self-reported clinical measures as possible predictors of WM status of FM patients are summarized in Table 4 (only variables with significant coefficients are shown).

**TABLE 4:** Results of the linear regression analysis for working memory measures.

	Model overview				Predictive variable	Linear regression coefficients			*t*	*p*
	R	R^2^	Adjusted R^2^	Standard error		Not standardized		Standardized		
						B	Standard error	Beta		
2-Back Correct	0.449	0.201	–0.009	9.097	VAS: Fatigue	1.786	0.739	0.366	2.417	0.018
Digit Span: Forward	0.611	0.373	0.231	1.921	VAS: Non-restorative Sleep	0.234	0.116	0.273	2.020	0.047
Digit Span: Forward Span	0.556	0.309	0.152	1.165	VAS: Non-restorative Sleep	0.157	0.070	0.317	2.237	0.028
Digit Span: Backward	0.497	0.247	0.077	1.727	−	−	−	−	−	−
Digit Span: Backward Span	0.448	0.201	0.020	1.097	−	−	−	−	−	−
Spatial Span: Forward	0.441	0.195	0.013	1.922	−	−	−	−	−	−
Spatial Span: Forward Span	0.469	0.220	0.044	1.127	FIQ-R: Function	–0.073	0.034	–0.392	–2.146	0.035
Spatial Span: Backward	0.596	0.356	0.210	1.636	VAS: Fatigue	0.340	0.114	0.352	2.996	0.004
					VAS: Health status	–0.249	0.083	–0.351	–3.014	0.003
Spatial Span: Backward Span	0.535	0.287	0.125	1.014	VAS: Fatigue	0.189	0.070	0.332	2.687	0.009
					VAS: Health status	–0.140	0.051	–0.336	–2.745	0.007
					PSQI	0.070	0.033	0.282	2.086	0.040
ACT at 0 s	0.512	0.262	0.089	1.481	VAS: Health status	0.252	0.076	0.423	3.334	0.001
ACT at 9 s	0.558	0.312	0.150	2.967	Pain Threshold	–2.122	1.039	–0.665	–2.042	0.044
					Pain Tolerance	1.279	0.626	0.583	2.043	0.044
ACT at 18 s	0.481	0.232	0.052	3.450	FIQ-R: Symptoms	–0.154	0.076	–0.395	–2.041	0.045
ACT at 36 s	0.386	0.149	–0.051	3.547	–	−	−	−	−	−
Total ACT Score	0.499	0.249	0.072	8.029	–	−	−	−	−	−

*VAS, Visual Analogical Scale; FIQ-R, Fibromyalgia Impact Questionnaire; ACT, Auditory Consonant Trigram Test.*

Linear regression analysis indicated that sleep problems predict patient performance on some of the WM scores. The non-restorative sleep VAS yielded significant coefficients for the Digit Span Forward task, both for its direct (*R*^2^ = 0.373; β = 0.273; *t* = 2.020; *p* = 0.047) and span (*R*^2^ = 0.309; β = 0.317; *t* = 2.237; *p* = 0.028) scores. In addition, the PSQI was able to predict performance on the Spatial Span Backward span score (β = 0.282; *t* = 2.086; *p* = 0.040).

The results also showed the predictive capacity of the VAS-fatigue for several WM scores, specifically for the 2-Back correct score (β = 0.366; *t* = 2.417; *p* = 0.018), and for both Spatial Span Backward direct (β = 0.352; *t* = 2.996; *p* = 0.004) and span scores (β = 0.332; *t* = 2.687; *p* = 0.009).

In addition, the Health Status VAS showed predictive capacity on the Spatial Span Backward task, both for the direct (*R*^2^ = 0.356; β = −0.351; *t* = −3.014; *p* = 0.003) and span (*R*^2^ = 0.287; β = −0.336; *t* = −2.745; *p* = 0.007) scores, and on the 0 s ACT score (*R*^2^ = 0.262; β = 0.423; *t* = 3.334; *p* = 0.001).

Regards to pain measures, both Pain Threshold (β = −0.665; *t* = −2.042; *p* = 0.044) and Pain Tolerance (β = 0.583; *t* = 2.043; *p* = 0.044) yielded significant coefficients for the 9 s ACT score (*R*^2^ = 0.312).

Finally, the Function domain of FIQ-R yielded significant coefficients for the Spatial Span Forward span score (*R*^2^ = 0.220; β = −0.392; *t* = −2.146; *p* = 0.035), and the Symptoms domain yielded significant coefficients for the 18 s ACT score (*R*^2^ = 0.232; β = −0.395; *t* = −2.041; *p* = 0.045).

Neither depression, anxiety nor subjective complaints seemed to be related to or to predict patients’ performance in the different WM tasks.

## Discussion

Although it is very common for patients with FM to report cognitive problems (Dick et al., 2008; Seo et al., 2012; Tesio et al., 2014; Coppieters et al., 2015; Gelonch et al., 2018), it is not yet clear how their cognitive functioning is related to alterations in pain processing. Some authors have hypothesized that an overlap between nociceptive and working memory (WM) networks (Buhle and Wager, 2010) and an unequal distribution in the neural resources associated with chronic pain would lead to a decrease in the resources dedicated to cognitive tasks (Schmidt-Wilcke et al., 2014). However, there is not enough evidence to support this hypothesis. Likewise, it is not known exactly what other clinical variables could explain or be related to cognitive status. In the present study, we assessed the performance in different WM tasks and pressure pain thresholds in a large sample of patients with FM, to examine the relationship between their cognitive performance and pain perception. Furthermore, we evaluated the main symptoms of FM through self-reported tests, with the aim of determining their association with WM performance.

In this research, we attempted to obtain a general picture of the WM status of patients with FM by means of a comprehensive evaluation of all WM components. After comparing to normative data, we found a subtle WM impairment. Patients with FM seem to preserve their WM span and ability to maintain and manipulate information for both visuospatial and verbal domains, as indicated by the span scores on the Digit Backward and Spatial Backward tasks, but the percentage of FM patients impaired for the ACT task at 36 s, related to short-term memory, divided attention, and information processing capacity, reached the 36.43%. Findings are in line with previous studies reporting cognitive deficits in different areas of memory, such as short-term (Leavitt and Katz, 2006; Roldán-Tapia et al., 2007; Gelonch et al., 2018), long-term (Park et al., 2001; Roldán-Tapia et al., 2007; Tesio et al., 2014), and working-memory (Park et al., 2001; Dick et al., 2008; Cánovas et al., 2009; Kim et al., 2011; Seo et al., 2012; Tesio et al., 2014; Coppieters et al., 2015; Gelonch et al., 2018; Pidal-Miranda et al., 2018; Ferrera et al., 2020); however, they contrast with other studies that did not found altered information processing in patients with FM (Park et al., 2001). This discrepancy could be explained by the fact that WM performance is particularly affected in those tasks that entail a higher workload (Khaksari et al., 2019).

On the other hand, although the difference between the performance in the normal range on the most cognitive tasks and the high scores on the subjective memory complaints scale—with more than 50% of subjects scoring Moderate impairment—could be seen as striking, it actually reflects the nature of dyscognition in FM patients, which comprises both objective and subjective alterations. This dissociation has been explained based on a social desirability effect of patients with FM in the neuropsychological assessment (Stodel, 2015), which would lead to a better performance than in their day-to-day life. This possible masking of cognitive alterations -whether conscious or not- highlights the interest of studying the relationship between FM, its associated variables, and cognitive functioning.

We also attempted to understand the relationship between pain sensitivity and WM performance, based on the fact that several brain areas (insular cortex, cingulate cortex, supplementary motor area, hippocampal regions, etc.) are involved in both nociceptive processing and cognitive functioning. Previous studies reported that the detection and processing of nociceptive information can influence WM ability (Munguía-Izquierdo et al., 2008; Buhle and Wager, 2010; Sanchez, 2011; Moore et al., 2012; Hood et al., 2013; Nakae et al., 2013; Coppieters et al., 2015; Sturgeon et al., 2015; Boselie et al., 2016). We tested whether patients who experience higher sensitivity to nociceptive stimulation (lower pain threshold) would perform poorly in the WM tasks. Our data indicate only a correlation between pain threshold and ACT task, while other studies did not find any type of association (Suhr, 2003; Etherton, 2014; Gálvez-Sanchez et al., 2018). Furthermore, when performing Bayesian analyses, the evidence found also pointed to an absence of correlation between pain threshold and WM tasks, even for ACT. Likewise, lack of differences between patients with high and low pain threshold in any of the WM tasks does not support the hypothesis of an overlap between pain and cognition networks as an explanation for the cognitive dysfunction in FM (Buhle and Wager, 2010; Schmidt-Wilcke et al., 2014; Sturgeon et al., 2015). Previous studies reported morphological (Luerding et al., 2008; Cánovas et al., 2009; Robinson et al., 2011; Ceko et al., 2012), functional (Napadow et al., 2010; Glass et al., 2011; Seo et al., 2012; Schmidt-Wilcke et al., 2014; González-Villar et al., 2017) and neurotransmission alterations in the dopaminergic (Albrecht et al., 2015; Ferrera et al., 2020), glutamatergic and GABAergic systems (De Paepe et al., 2020), which could be associated with pain in patients with FM. Future studies should clarify whether the WM functioning in FM can be explained by pain-mediated structural and functional neural reconfiguration.

Finally, we attempted to examine which of the clinical self-reported variables were the best predictors of WM performance. The results of the linear regression analyses suggest that sleep problems and fatigue were the variables that best predicted WM performance in FM patients, as found in previous research (Suhr, 2003; Ceko et al., 2012; Aasvik et al., 2018; Pidal-Miranda et al., 2018; Andrade et al., 2020). In addition, both pain threshold and pain tolerance appear to predict performance on the ACT task at 9 s, as already indicated by the correlation analysis. Similarly, health status seems to predict both the spatial span and ACT tasks.

In the case of sleep problems, unrefreshing sleep assessed by VAS was a significant predictor of direct and span scores in the verbal WM forward task, and the score obtained in the Pittsburgh Sleep Quality Index was significantly related to the span in the visuospatial WM backward task. These findings are consistent with those of previous studies supporting the idea that insomnia can negatively impact the WM of participants, even if they have not a diagnosis of FM (Aasvik et al., 2018). However, other studies suggest that alterations in cognitive performance may persist even when patients’ sleep problems are controlled for Dick et al. (2008). Although FM patients report a high prevalence of sleep problems (Anderson et al., 2012; Andrade et al., 2020; Mun et al., 2020; Türkoğlu and Selvi, 2020), the neurobiological characteristics underlying these problems and their relationship to nociceptive and cognitive impairment have not yet been fully investigated (Ceko et al., 2012).

On the other hand, VAS-fatigue proved to be a significant predictor of performance in the 2-Back task and of direct and span scores of the Spatial Span Backward task. This is consistent with previous findings (Suhr, 2003; Pidal-Miranda et al., 2018), which indicated that the fatigue experienced by patients with FM can influence their poor performance on WM tasks, or their performance perception (Williams et al., 2011). However, other authors found that cognitive problems can appear in the absence of fatigue (Dick et al., 2008).

In this study, we did not find any relationship between cognitive performance in FM patients and the clinical and self-reported scales used to assess anxiety and depression (HADS, BDI-1A, Mood VAS), and therefore we cannot corroborate results obtained in other studies on chronic pain syndromes (Williams et al., 2011; Gelonch et al., 2013, 2016, 2017, 2018; Walitt et al., 2016; Pidal-Miranda et al., 2018).

The study findings also failed to confirm any relationship between subjective cognitive complaints and objective WM performance in the FM patients. This is consistent with the results of previous studies suggesting a disconnection between neuropsychological performance and subjective complaints in FM patients (Gelonch et al., 2016; Walitt et al., 2016; Pidal-Miranda et al., 2018). It is also consistent with studies showing altered brain activity in FM patients performing WM tasks, even in the absence of behavioral impairment (Luerding et al., 2008; Glass et al., 2011; Ceko et al., 2012; Seo et al., 2012; Schmidt-Wilcke et al., 2014; Walitt et al., 2016; González-Villar et al., 2017).

Regarding the limitations of this study, several factors could affect the generalizability of the results obtained. On the one hand, the sample used was composed exclusively of women with FM because the prevalence of FM in the Spanish population is much higher in women (4.2%) than in men (0.2%) (Mas et al., 2008). In addition, in this way the sample was more homogeneous, and we were able to control gender differences. However, recent studies indicate that previous assumptions of the higher prevalence and symptom severity (i.e., sleep problems) of FM in women should be reconsidered (Segura-Jiménez et al., 2016; Sallinen and Mengshoel, 2018). Therefore, although no significant symptom differences have been found in relation to gender (Segura-Jiménez et al., 2016), extrapolation of our results to male patients should be done with caution. In addition, the established age criterion was very broad. Future research should include men and should control sociodemographic variables such as patients age, to enable generalization of the findings.

Finally, the pain threshold measurement used is static in nature. As one of our objectives was to investigate the relationship between WM performance and pain experience, it might have been more appropriate to use dynamic indices, such as those obtained in the Conditioned Pain Modulation protocol (CPM; Yarnitsky, 2010). The CPM assesses endogenous mechanisms of analgesia based on the reduction or increase in the pain threshold at a point produced by nociceptive stimulation on another remote location; a recent meta-analysis study suggests that this could be a useful protocol for the study of nociceptive alterations in FM (O’Brien et al., 2018). Regarding future research, we think it would be interesting to perform the CPM protocol simultaneously to the execution of a 2-Back task. It would also be of interest to examine whether WM activity interferes with nociceptive processing and modulation or if the induced pain affects the performance of the 2-Back task, as indicated in recent attentional studies (Moore et al., 2019).

## Conclusion

Patients with fibromyalgia seem to preserve their ability to process and manipulate information in working memory but show impairment in tasks requiring more short-term memory load, divided attention, and information processing ability. Contrary to the expected results, pain threshold was only related to performance in the more demanding WM tasks (ACT task). Among the clinical variables associated with FM, sleep problems and fatigue seem to be the most closely related to cognitive functioning.

## Data Availability Statement

The raw data supporting the conclusions of this article will be made available by the authors, without undue reservation.

## Ethics Statement

The studies involving human participants were reviewed and approved by Research Ethics Committee of Galicia (code: 2014/488). The patients/participants provided their written informed consent to participate in this study.

## Author Contributions

MP-M, DR-S, NS-V, and MC designed the study. MP-M and NS-V recruited the participants and made the assessments. AG-U, DR-S, MF-P, and MC analyzed and interpreted the data. AG-U wrote the first draft of the manuscript. AG-U, DR-S, and MC reviewed and wrote the last version of the manuscript. All authors contributed to the article and approved the submitted version.

## Conflict of Interest

The authors declare that the research was conducted in the absence of any commercial or financial relationships that could be construed as a potential conflict of interest.

## Publisher’s Note

All claims expressed in this article are solely those of the authors and do not necessarily represent those of their affiliated organizations, or those of the publisher, the editors and the reviewers. Any product that may be evaluated in this article, or claim that may be made by its manufacturer, is not guaranteed or endorsed by the publisher.
